# Prediction and variability mapping of some physicochemical characteristics of calcareous topsoil in an arid region using Vis–SWNIR and NIR spectroscopy

**DOI:** 10.1038/s41598-022-12276-4

**Published:** 2022-05-19

**Authors:** Samer Alomar, Seyed Ahmad Mireei, Abbas Hemmat, Amin Allah Masoumi, Hossein Khademi

**Affiliations:** 1 Department of Biosystems Engineering, College of Agriculture, Isfahan University of Technology, Isfahan, 84156-83111 Iran; 2 Department of Soil Science, College of Agriculture, Isfahan University of Technology, Isfahan, 84156-83111 Iran

**Keywords:** Chemistry, Engineering, Mathematics and computing, Optics and photonics

## Abstract

Site-specific management of soils needs continuous measurements of soil physicochemical characteristics. In this study, Vis–NIR spectroscopy with two spectroscopic instruments, including charge-coupled device (CCD) and indium-gallium-arsenide (InGaAs) spectrometers, was adopted to estimate some physicochemical characteristics of a calcareous topsoil in an arid climate. Partial least squares (PLS) as linear and artificial neural networks (ANN) as nonlinear multivariate techniques were utilized to enhance the accuracy of prediction. The best predictive models were then used to extract the variability maps of physicochemical characteristics. Diffuse reflectance spectra of 151 samples, collected from the calcareous topsoil, were acquired in the visible and short-wavelength near-infrared (Vis–SWNIR) (400–1100 nm) and near-infrared (NIR) (950–1650 nm) spectral ranges using CCD and InGaAs spectrometers, respectively. The results showed that NIR spectral data of the InGaAs spectrometer was necessary to reach the best predictions for all selected soil properties. The best predictive models based on the optimum spectral range could allow us the excellent predictions of sand (RPD = 2.63) and silt (RPD = 2.52), and very good estimations of clay (RPD = 2.35) and electrical conductivity (EC) (RPD = 2.224) by ANN and very good prediction of calcium carbonate equivalent (CCE) (RPD = 2.01) by PLS. The CCD device, however, resulted in acceptable predictions of sand (RPD = 2.13, very good) and clay (RPD = 1.66, fair) by ANN, and silt (RPD = 1.78, good), EC (RPD = 1.84, good) and CCE (RPD = 1.67, fair) by PLS. Similar variability was attained between pairs of predicted maps by best models and reference-measured maps for all studied soil properties. For clay, sand, silt, and CCE, the Vis/SWNIR-predicted and equivalent reference-measured maps had acceptable similarities, indicating the potential application of low-cost CCD spectrometers for prediction and the variability mapping of these parameters.

## Introduction

Soil is one of the major natural resources that has evolved through weathering processes (climatic, geologic, and biological activities) on parent material. The soil is characterized by a variety of spatial properties. Site-specific management practices provide satisfactory environmental and economic impacts on agricultural production. However, these practices need to continuously measure and recognize the temporal and spatial variations of physicochemical soil properties. Previously, these properties were determined using conventional methods that require collecting a sufficient number of soil samples to include all the spatial variations in the farm, a lot of work, and expensive chemical materials. Therefore, these methods cannot be applied in site-specific measurements and variable-rate application of inputs in farms^1–3^.

In recent years, researchers have tended to discover an alternative method for conventional laboratory measurements of physicochemical soil properties. Visible and near-infrared (Vis–NIR) spectroscopy has been adopted as one of these alternative methods. This method can rapidly conduct the soil analysis and hence it can be used for managing the spatial variation of the soil properties with high-resolution^1,4^. Vis–NIR spectroscopy is divided into field- and lab-based spectroscopy. In lab-based spectroscopy, conditions of spectroscopic measurements and samples are controlled by removing heterogeneous soil structure and illumination change. While, field-based spectroscopy is influenced by many factors such as variable-size soil aggregates, raw organic residues, and heterogeneous soil surfaces, and changes in soil moisture, distance between soil and probe sensor, and illumination. Therefore, the results obtained from the field-based spectroscopy are usually less accurate than those obtained from the lab-based spectroscopy^5,6^.

Different physicochemical properties of soil have been successfully estimated by Vis–NIR spectroscopy. These include clay, sand, silt, and electrical conductivity (EC) as well as nutritional soil properties such as organic matter (OM), total nitrogen (TN), available potassium (K_-avl_), and available phosphorus (P_-avl_)^5,7,8^. Table 1 summarizes a review of some recent studies that used lab-based Vis–NIR spectroscopy for estimating the selected physicochemical properties of different soil types. There is a growing demand to quantitatively determine the quality and spatial variability of calcareous soils in arid climates to recognize and improve soil condition and crop productivity and to use variable-rate inputs application. As shown in Table 1, to the best of our knowledge, no studies have so far been conducted to estimate physicochemical properties of calcareous soils in arid climates using lab-based Vis–NIR spectroscopy. Furthermore, as shown in Table 1, most researchers have preferred to use the full spectroscopic range of the NIR region, i.e., 780–2500 nm. Economically, full-range and even part-range NIR spectrometers with the spectral range of 900–1700 nm, are remarkably more expensive than the visible and short-wavelength near-infrared (Vis–SWNIR) spectrometers with a more narrow spectral range of 400–1100 nm. Among recent literature, no comparison has been made on the cost and the prediction accuracy of selected soil properties between these spectrometers.

**Table 1:** A review of recent literature that used lab-based Vis–NIR spectroscopy for estimating some physicochemical properties of soil.

Reference	Soil type	spectroscopic range (nm)	Sample treatment	Soil properties	Models	*R*^2^_p_	RMSEP	RPD
Summers, et al.^32^	Chromosols, Dermosols, Rudosols, and Kurosols	400–2500	oven-dried sieved 2 mm	Clay	PLS	0.66	3.13^g^	2.0
				Carbonate	PLS	0.69	2.90^g^	2.1
Tümsavaş, et al.^1^	Clay and clay loam	350–2200	fresh	Clay	PLS	0.91	2.67^g^	3.51
				Sand	PLS	0.90	2.91^g^	3.25
Wetterlind, et al.^31^	Glacial and postglacial clay	350–2500	air-dried sieved < 2 mm	Clay	PLS	0.75–0.95	3.6^g^–2.7^g^	2.3–3.7
				Silt	PLS	0.63–0.73	3.4^g^–2.8^g^	1.5–1.8
				Sand	PLS	0.91–0.93	3.8^g^–2.5^g^	3.3–3.4
Debaene, et al.^7^	Podzoluvisol	400–2200	air- dried sieved 2 mm	Clay	PLS	0.73	0.32^g^	1.9
				Silt	PLS	0.79	2.25^g^	2.2
				Sand	PLS	0.79	2.52^g^	2.2
Feyziyev, et al.^33^	Soilsare Calcisols, Solonchaks, and Calcaric Fluvisols	350–2500	air-dried sieved 2 mm	EC	PLS	0.82	0.09^h^	–
				CaCO_3_	PLS	0.90	2.90^i^	–

*CaCO*_*3*_ calcium carbonate, *EC* electrical conductivity, *PLS* partial least squares, *R*^2^_p_ coefficient of determination in prediction, *RMSEP* root mean square error of prediction (^g^ %, ^h^standard error dS.m^−1^, ^i^standard error g.kg^−1^),* RPD* residual prediction deviation.

Due to the low values of some soil parameters, absorption interference can occur in the NIR spectrum. Therefore, Vis–NIR spectra of soil samples are relatively weak and non-specific and it would be difficult to distinguish spectral patterns of these components. Special techniques are needed to obtain these spectral patterns and make a successful correlation between these patterns and selected soil properties^9,10^. These techniques include the appropriate selection of spectrometer, light source, optical probe^11^, and multivariate calibration method^12^, control of surrounding conditions^13^, and use of suitable pretreatment of the spectroscopic data^14^. Hence, the appropriate selection of multivariate calibration technique, and the ability to interpret spectroscopic data are considered the key steps for improving the estimation accuracy of soil properties^15,16^. Linear multivariate calibration models such as partial least squares (PLS) regression have been efficient to extract mathematically complex spectral patterns and find the correlation between these patterns and the soil properties. However, due to the nonlinear relationship between spectroscopic data and soil properties, there has been a growing interest in using nonlinear multivariate calibration methods such as artificial neural networks (ANN) regression^9,10^.

The aims of the present research were: (1) to evaluate the potential of the lab-based Vis–NIR spectroscopy for estimating soil physicochemical properties including clay, sand, silt, calcium carbonate equivalent (CCE), and EC in restructured soil samples, collected from the calcareous topsoil in an arid climate, (2) to study the ability of two spectroscopic instruments including a high-cost InGaAs with NIR spectroscopic range (950–1650 nm), and a low-cost CCD with Vis–SWNIR spectroscopic range (400–1100 nm) for estimating selected physicochemical properties of soil, and (3) to compare the performance of linear (PLS) and non-linear (ANN) multivariate calibration methods to estimate the selected soil physicochemical properties. In general, prediction of various physicochemical properties (clay, sand, silt, CCE, and EC) of selected soil type (calcareous topsoil) in an arid climate, and comparison of two spectroscopic instruments (InGaAs and CCD) along with the utilization of different multivariate calibration methods could be considered as the novelty aspects of the present research.

## Materials and methods

### Study farm

The soil samples were collected from the Lavark research farm (32°32′N, 51°23′E) of the Isfahan University of Technology, located in Isfahan province, Iran. The farm has an arid climate with average annual precipitation of 140 mm, a mean temperature of 14.5 °C, and an altitude of 1630 m above sea level. The soil is a fine-loamy calcareous that developed on alluvial deposits of the Zayandehroud River. It has a history of conventional tillage operations, and low organic matter (less than 0.5%). However, during the last 6 years, decayed farmyard manure has been uniformly applied to the soil surface at a rate of 50 Mg ha^-1^, then mixed to 15 cm depth of topsoil using tillage practices. The crop rotation was irrigated maize silage-barley during the last 6 years.

### Sampling and preparation for spectroscopy

A total of 151 composite soil samples were collected from an area of 32 ha, after harvesting barley, with a rate of one sample per 2000 m^2^ plot. The detailed information about the area of study and soil sampling locations can be found in our previous study^8^ in which the soil fertility parameters of the same points were investigated. Each composite sample was obtained from 5 subsamples taken from the center and corners of the square with a side length of 5 m. The samples were separately stored in plastic bags and transported to the laboratory. For reducing the effect of soil structure on the accuracy of the estimation and getting homogeneous samples, air-drying and sieving to 2 mm were adopted, before spectra acquisition and reference analyses.

### Spectra acquisition

Two spectroscopic instruments were adopted to record the diffuse reflectance spectra from the horizontal and smooth surfaces of soil samples. The first instrument was a CCD-based Vis–SWNIR (model: LR1 spectrometer, ASEQ Instrument, Vancouver, Canada), with a wavelength range of 200–1200 nm and a wavelength resolution of approximately 0.2 nm. However, due to the light sources utilized in this study (tungsten-halogen lamps) and the noise of the spectrometer, the spectral range of 400–1100 nm was used for further analysis. Two tungsten-halogen lamps (50-W) were utilized as the light sources in the diffuse reflectance mode and placed at an angle of 45° to the horizontal line above a Petri dish (a depth of 10 mm and a diameter of 100 mm) containing 30 g of each soil sample. The horizontal distance between light sources and the center of the Petri dish was 12 cm. A bundle fiber optic probe (R600-8 Vis–NIR, StellarNet, Inc. Oldsmar, Florida, USA) was utilized to collect the diffused reflection lights from the surface of the soil sample and pass them to the spectrometer. This probe was placed in a vertical position at a distance of 10 mm and an angle of 90° to the Petri dish. The exposure time and the number of scans were selected as 50 ms and 10, respectively. Three replicate spectra were collected from each soil sample by 120° rotating of the Petri dish and the average spectrum was calculated and used for the subsequent calculations. To obtain the relative spectra, the reference and dark measurements were carried out at the beginning of each 15 spectra readings. Finally, reflectance spectra (*R*) were converted to absorbance spectra (*A*) by adopting the equation of *A* = log (1/*R*).

The second spectroscopic instrument was a commercial NIR device (DA 7250™ NIR analyzer, Perten Instruments, PerkinElmer Inc., USA). The instrument was equipped with a photodiode array NIR spectrometer with a thermoelectrically cooled 256 element InGaAs detector, capable to acquire spectral data in a wavelength range and resolution of 950–1650 nm and 0.5 nm, respectively. Each spectrum consisted of an average of 10 scans collected via automatic rotating of the sample dish. Using a Teflon-coated ceramic reference flag in the instrument, the reference and dark spectra were automatically measured.

### Reference analysis

To create models and evaluate them through comparing the estimated and reference values, the selected soil physicochemical properties including clay, sand, silt, CCE, and EC were measured using standard laboratory methods. The pipette method^17^ was used to determine clay, sand, and silt contents, and the titration method^18^ and soil–water suspension (1:2)^19^ were adopted to measure the CCE and EC, respectively.

### Spectroscopic data analysis

Before spectroscopic data analysis, data were reviewed and sample outliers were detected. Principal components analysis (PCA) was first performed to identify the potential outliers in spectral data (or X-variables)^20^. Samples situated outside the Hotelling T^2^ ellipse with the 95% confidential level were identified as the outliers and removed from the data set. Then, by performing PLS analysis, the plot of residual sample variance for Y-variables (measured soil properties) was checked to detect the possible outliers due to errors in reference measurements. For each parameter, the samples with the extreme value of sample residual were detected as the outlier and eliminated from the data set. 6 samples were detected as the outlier in the spectral data after performing the PCA. Additionally, 4, 2, 2, 2, and 0 samples were identified as the outlier with extreme Y-residuals in clay, sand, silt, CCE, and EC data sets, respectively. The remaining samples were then randomly divided into two subsets, including the calibration (80% of total samples) and the independent validation or test (20% of remaining samples) subsets. The calibration subset was adopted for developing predictive models, while the test subset was utilized to assess the robustness of developed models.

Before developing spectroscopic models, averaging and Savitsky-Golay (SG) smoothing were applied to the spectral data to enhance the spectroscopic features and signal-to-noise ratio^21^. The number of averaging and smoothing points for each studied physicochemical property was optimized using the trial and error procedure. Different pre-processing techniques were then applied to remove unwanted and irrelevant information from spectroscopic data. These techniques included standard normal variate (SNV), multiplicative scatter correction (MSC), linear baseline correction (LBC), baseline offset elimination (BOE), minimum, maximum, range mean, area, and unit vector normalization, SG first and second derivatives (1st Der. and 2nd Der.), and each combination of two techniques. For each studied soil property, the best pre-processing technique, which resulted in the best predictability of the PLS model, was selected and adopted for further spectroscopic analyses.

PLS and ANN multivariate techniques were used to extract predictive models of selected soil physicochemical properties, and their performances were then compared. The spectral data of CCD and InGaAs spectrometers and the combination of both (with the range of 400–1650 nm) were used to develop the PLS and ANN predictive models. The performance of spectrometers and the data combination of both were then compared to reach the maximum predictability of each studied soil property. The PLS and ANN methods are briefly described below, and for more details, the references were cited.

The PLS regression is based on disassembling the spectroscopic data into the features named latent variables (LV), which include most of the variance in the spectroscopic data along with the variance of the response variables as much as possible^20,22^. To increase the covariance between predictors (wavelengths) and response variables (selected properties), the PLS algorithm integrates the compression and regression steps, leading to identifying consecutive orthogonal factors, i.e., LVs^5^. The leave-one-out cross-validation (LOOCV) was adopted for evaluating the PLS models for the calibration set^23^. By testing a plot of the LOOCV residual variance versus the number of LVs, the optimal number of LVs for each PLS model was selected^24^. By applying the regression coefficients (b-coefficients), the effective wavelengths in the PLS calibrations were specified. When the b-coefficient value of a specific wavelength exceeds the standard deviation of all b-coefficient values, the corresponding wavelength is considered effective^22,25,26^. After developing the PLS models for selected soil physicochemical properties, the model’s predictability was evaluated by an independent validation subset of samples.

In ANN regression, a multilayer feed-forward neural network with the back-propagation (BP) learning algorithm was adopted for estimating the selected soil physicochemical properties. This type of network (BPANN) is the most appropriate and prevalent architecture for modeling complex nonlinear spectroscopic data, because of its supervised learning ability. The BP is the generalization of the Widrow–Hoff learning rule for training the multilayer perceptron (MLP) networks with nonlinear transfer functions. By calculating the case-wise error function, the overall network error was reduced^27^. For each soil property, a three-layer perceptron network (input, output, and hidden layer) was used. However, due to the large size of the spectroscopic data, PCA was first applied to them and the PCA-vectors (PCs) were adopted as the input of the neural network. It caused reducing the mathematical operations and enhancing the robustness of the models^15^. The trial and error procedure was applied to define the optimal number of neurons in the hidden layer and the best pair of activation functions in the hidden and output layer. The network topologies with the maximum predictability of soil properties were selected. Moreover, three different training algorithms of gradient descent (GD), conjugate gradient (CG), and Broyden–Fletcher–Goldfarb–Shanno (BFGS) were examined for each network architecture to reach the maximum estimation accuracy.

Pre-processing the spectroscopic data, removing outliers, PCA, and PLS procedures were all performed by using the ‘The Unscrambler’ V10.4 software (CAMO AS, Trondheim, Norway). The ANN regression was carried out using ‘STATISTICA’ V12 (StatSoft, Inc., CA, USA). Finally, the spatial variability mapping was performed by using ‘ArcGIS’ V10.6.1.

The statistical parameters adopted for assessing the predictive power of models included the coefficient of determination in prediction (*R*^2^_p_), root mean squares error of prediction (RMSEP), and residual predictive deviation (RPD). The RPD is determined as the standard deviation of the measured values of the independent validation subset divided by the RMSEP^28^. The model performance was evaluated according to the criterion presented by Rossel, et al.^23^. The RPD > 2.5 refers to the excellent prediction, RPD = 2–2.5 indicates very good ability of model, RPD = 1.8–2.0 allows the good predictability, RPD = 1.4–1.8 results in fair predictive model, RPD = 1.0–1.4 refers to the poor estimation, and RPD < 1.0 shows a very poor prediction.

## Results and discussion

### Descriptive statistics of the soil properties

**Figure 1:**
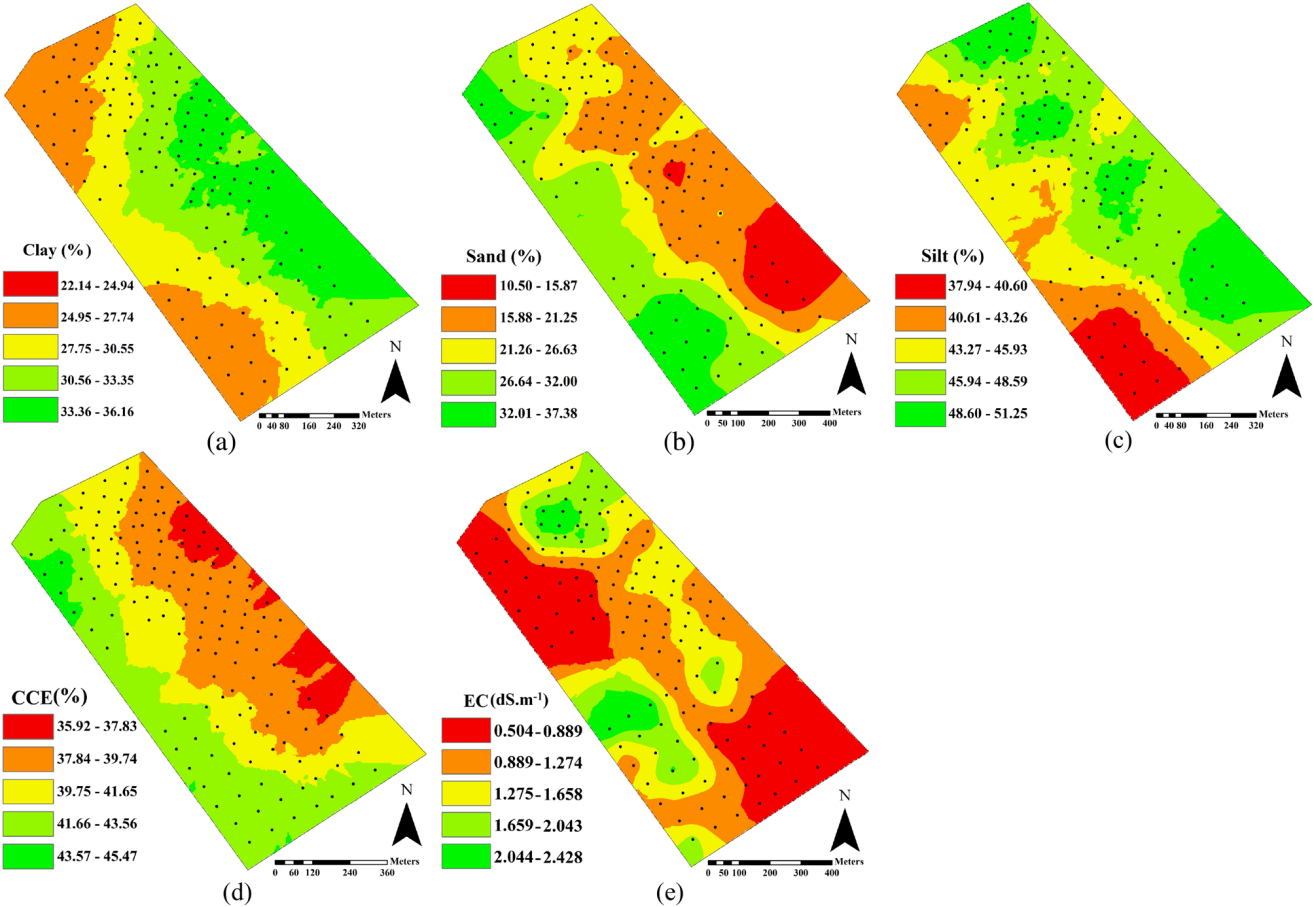
Spatial variation of reference-measured values of selected soil physicochemical properties (**a**) clay, (**b**) sand, (**c**) silt, (**d**) calcium carbonate equivalent (CCE), and (**e**) electrical conductivity (EC) in the studied farm scale.

The descriptive statistics of soil physicochemical properties obtained from reference measurements for the total, calibration, and independent validation subsets are provided in Table 2. Based on the coefficient of variation (%CV) values and the criterion presented by Wilding^29^, the variation was evaluated as high for EC (CV = 51.24%), moderate for sand (CV = 31.78%), and low for clay, silt and CCE (CV = 12.81%, 10.44% and 6.28%, respectively). In this research, variations in soil physicochemical properties are likely due to the differences in parent material, surficial deposits, agricultural operations (e.g., land leveling and tillage), planting patterns, irrigation, and harvest of the crop (residue and yield management). Figure 1 shows the spatial variation of the selected soil physicochemical properties within the studied farm scale. As shown, the soil properties were spatially variable within the studied farm. The wide range and enough spatial variability among studied properties could satisfy the prerequisite for obtaining acceptable predictive models in using Vis–NIR spectroscopy^30^. As expected, the variability map of clay content (Fig. 1a) had an opposite spatial distribution pattern to that for sand content (Fig. 1b).

**Table 2:** Summary of descriptive statistics values for the total, calibration, and validation reference data subsets of the selected soil physicochemical properties.

Soil properties	Sample subsets	Min	Max	Mean	SD	%CV
Clay (%)	Total (n = 141)	19.20	40.80	30.40	3.90	12.81
	Calibration (n = 113)	19.20	38.40	30.24	3.82	12.64
	Validation (n = 28)	24.00	40.80	31.06	4.18	13.47
Sand (%)	Total (n = 143)	9.44	42.36	22.94	7.29	31.78
	Calibration (n = 115)	9.44	41.80	23.04	7.30	31.67
	Validation (n = 28)	9.90	42.36	22.57	7.39	32.75
Silt (%)	Total (n = 143)	34.06	56.50	46.06	4.81	10.44
	Calibration (n = 115)	34.20	56.14	46.17	4.63	10.03
	Validation (n = 28)	34.06	56.50	45.60	5.55	12.16
CCE (%)	Total (n = 143)	34.50	47.00	40.09	2.52	6.28
	Calibration (n = 115)	34.50	47.00	40.07	2.52	6.29
	Validation (n = 28)	36.50	46.50	40.18	2.55	6.36
EC (dS.m^-1^)	Total (n = 145)	0.340	2.780	1.205	0.617	51.24
	Calibration (n = 116)	0.340	2.560	1.190	0.605	50.79
	Validation (n = 29)	0.487	2.780	1.262	0.674	53.43

*CCE* calcium carbonate equivalent, *CV* coefficient of variation, *EC* electrical conductivity, *Max*. maximum, *Min.* minimum, *SD* standard deviation.

### Modeling by PLS

Table 3 summarizes PLS regression results of the soil physicochemical properties, including clay, sand, silt, CCE, and EC using spectroscopic data obtained from CCD and InGaAs spectrometers and the combination data of both spectrometers. The PLS regression presented various estimation accuracies of the selected soil properties when the spectral data from each spectrometer or both were used.

**Table 3:** Calibration and estimation PLS results of selected soil physicochemical properties using spectral data of CCD and InGaAs spectroscopic instruments, and the combination data of both.

Soil properties	Spectroscopic instrument	Preprocessing	Calibration subset			Test subset		
			Rank	*R*^2^_c_	RMSEC	*R*^2^_p_	RMSEP	RPD
Clay (%)	CCD	1st Der	7	0.761	1.86	0.651	2.58	1.62
	InGaAs	1st Der. + MSC	10	0.739	1.98	0.720	2.27	1.84
	Combination	LBC + BOE	5	0.695	2.10	0.717	2.14	1.95
Sand (%)	CCD	1st Der	7	0.793	3.31	0.762	3.54	2.09
	InGaAs	BOE	12	0.846	2.81	0.894	2.88	2.57
	Combination	RN	7	0.802	3.35	0.801	2.81	2.63
Silt (%)	CCD	1st Der	11	0.772	2.20	0.732	3.12	1.78
	InGaAs	BO	11	0.839	2.02	0.734	2.32	2.39
	Combination	2nd Der	5	0.518	3.39	0.572	3.52	1.58
CCE (%)	CCD	2nd Der	3	0.658	1.47	0.681	1.53	1.67
	InGaAs	MSC	5	0.668	1.49	0.876	1.27	2.01
	Combination	1st Der	9	0.743	1.28	0.450	1.81	1.41
EC (dS.m^-1^)	CCD	1st Der	6	0.790	0.276	0.695	0.366	1.84
	InGaAs	MEN	15	0.939	0.173	0.734	0.329	2.05
	Combination	1st Der	5	0.723	0.322	0.695	0.359	1.88

*BOE* baseline offset elimination, *CCD* charge-coupled device, *CCE* calcium carbonate equivalent, *EC* electrical conductivity, *InGaAs* indium gallium arsenide, *LBC* linear baseline correction, *MEN* mean normalization, *MSC* multiplicative scatter correction, *R*^2^_c_ coefficient of determination in calibration, *R*^2^_p_ coefficient of determination in prediction, *RMSEC* root mean square error of calibration, *RMSEP* root mean square error of prediction, *RN* range normalization, *RPD* residual predictive deviation, *1st Der.*: first derivative, *2nd Der.* second derivative.

**Figure 2:**
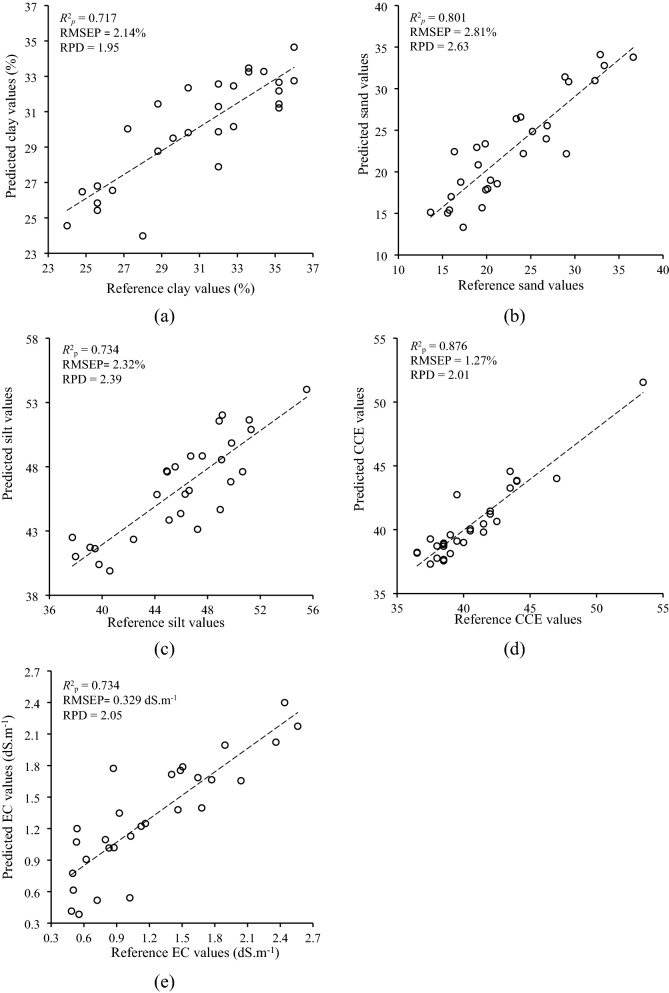
Reference versus estimated values obtained by the best PLS models in predicting (**a**) clay and (**b**) sand using the combination data of both instruments, and (**c**) silt, (**d**) calcium carbonate equivalent (CCE), and (**e**) electrical conductivity (EC) using spectral data of InGaAs spectrometer.

Among selected soil properties, clay and sand offered an accurate estimation with the full range of Vis–NIR (400–1650 nm). Based on the criterion presented by Rossel, et al.^23^, the predictability of the best clay (*R*^2^_p_ = 0.717; RMSEP = 2.14%; and RPD = 1.95) and sand (*R*^2^_p_ = 0.801; RMSEP = 2.81%; and RPD = 2.63) PLS models in independent validation subset displayed the good and excellent performance, respectively. However, the best silt (*R*^2^_p_ = 0.734; RMSEP = 2.32%; and RPD = 2.39), CCE (*R*^2^_p_ = 0.876; RMSEP = 1.27%; and RPD = 2.01), and EC (*R*^2^_p_ = 0.734; RMSEP = 0.329 ms; and RPD = 2.05) models had very good performance when the spectroscopic data of InGaAs spectrometer was only utilized. The reference versus estimated values of selected soil physicochemical properties obtained the best PLS models are presented in Fig. 2.

The performance of CCD and InGaAs spectrometers showed that the InGaAs instrument outperformed the CCD one in estimating all soil physicochemical properties. For chemical components of CCE and EC, this was an expected result because the spectral range of the InGaAs spectrometer (950–1650 nm) included more information about the chemical components of the soil. However, due to the correlation of texture parameters with the soil components that have direct spectral responses in the NIR region^1^, the InGaAs spectral data provided a pivotal role to reach the maximum accuracy for predicting these parameters. In addition, the visible range (400–750 nm) data, associated with soil color and organic matter, played a complementary role to NIR data to reach the maximum predictability of clay and sand. Despite the superiority of NIR data, the Vis–SWNIR data of the CCD spectrometer led to acceptable estimations using the PLS method. In this case, the sand estimation (RPD = 2.09) was evaluated to be very good, the predictability of EC (RPD = 1.842) was good, while, the clay (RPD = 1.62), silt (RPD = 1.78), and CCE (RPD = 1.67) estimations were assessed as fair.

In terms of the lab-spectroscopy with the same soil sample conditions (air-dried, and sieved to 2 mm), the accuracy of the present PLS models (Table 3) was comparable with results reported in the previous literature (Table 1). The performance of the PLS model in estimating clay (*R*^2^_p_ = 0.717, and RPD of 1.95) was slightly better than those reported by Debaene, et al.^7^ with *R*^2^_p_ = 0.73, and RPD = 1.9, and less accurate compared with that reported by Wetterlind, et al.^31^ with *R*^2^_p_ = 0.75–0.95, and RPD = 2.3–3.7. Regarding the sand, the predictability of the PLS model (*R*^2^_p_ = 0.801, and RPD of 2.63) was better than the findings of Debaene, et al.^7^ with *R*^2^_p_ = 0.79, and RPD = 2.2, and less accurate compared with the findings of Wetterlind, et al.^31^, and Tümsavaş, et al.^1^ with *R*^2^_p_ of 0.90–0.93, and RPD of 3.25–3.4. For silt, the estimation power (*R*^2^_p_ = 0.734, and RPD of 2.39) was outperformed by the PLS models reported by Wetterlind, et al.^31^, and Debaene, et al.^7^ with *R*^2^_p_ of 0.63–0.79, and RPD of 1.5–2.2. Regarding the CCE, the predictability of the PLS model (*R*^2^_p_ = 0.786, and RPD of 2.01) was slightly less accurate than those presented by Summers, et al.^32^, and Feyziyev, et al.^33^ with *R*^2^_p_ of 0.69–0.90, and RPD of 2.1. For EC, the predictability of the present PLS model (*R*^2^_p_ = 0.734, and RPD of 2.05) was less accurate than the results of Feyziyev, et al.^33^ with *R*^2^_p_ = 0.82. By close investigation of the studies resulted in better accuracies, it was found that these studies have often utilized the full spectral range of the NIR region (780–2500 nm) in which more overtones and combination absorption bands could be detected to increase the predictability of the developed models. For example, a direct spectral response of clay exists in the wavelength range of 2200–2300 nm that is beneficial for clay prediction and mineralogy^1^. At the next step, the main challenge was to improve the prediction power and perhaps compensate the weakness of the shorter spectral range by using nonlinear multivariate techniques.

#### Regression coefficients (b-values)

**Figure 3:**
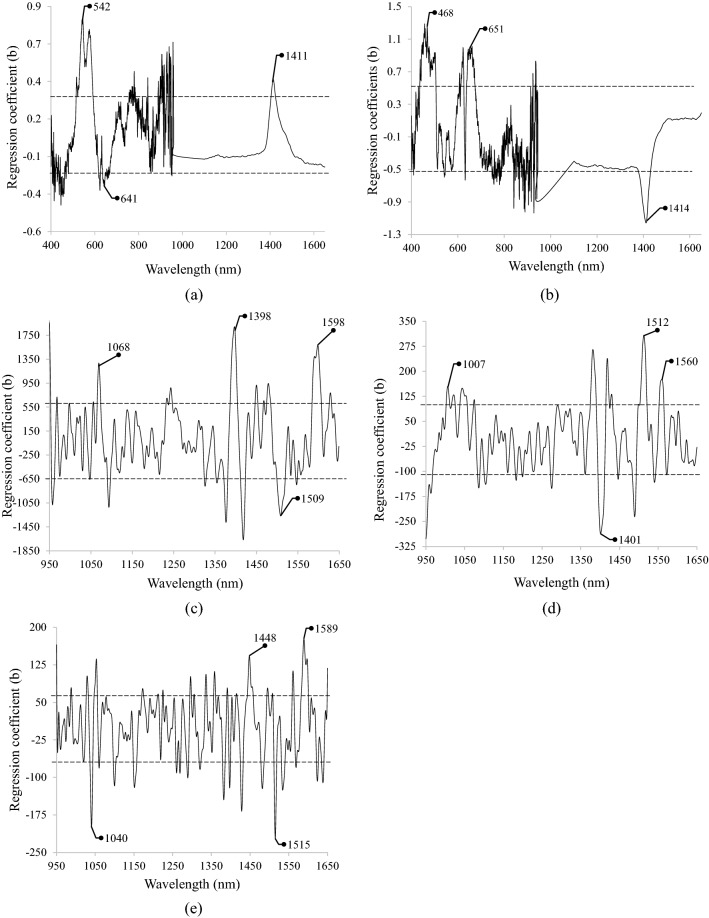
Regression coefficients (b-values) of the best PLS models for estimating (**a**) clay, (**b**) sand, (**c**) silt, (**d**) calcium carbonate equivalent (CCE), and (**e**) electrical conductivity (EC).

Figure 3 displays the regression coefficients (b-values) of the optimal spectral range obtained from the best PLS models for estimating selected soil physicochemical properties. The dashed lines represent the standard deviation of all b-values. The negative and positive b-values that exceeded the dashed line reveal the more efficient wavelengths in terms of explaining the variation in the selected property. For clay (Fig. 3a), the b-value curve had sharp positive peaks around 542 and 1411 nm and a negative peak at approximately 641 nm. For sand, the higher b-values were obtained close to 468, 651, and 1414 nm (Fig. 3b). For silt, the higher b-values were observed around 1068, 1398, 1509, and 1598 nm (Fig. 3c). Regarding CCE, wavelengths around 1007, 1401, 1512, and 1560 nm resulted in higher regression coefficients (Fig. 3d). Finally, for estimating EC the wavelengths around 1040, 1448, 1515, and 1589 nm were distinguished as important (Fig. 3e).

Regarding soil texture parameters, the important peak close to 542 nm for clay can be attributed to the chromophore (Fe-OOH) found in the goethite (a yellow mineral)^34^. About estimating sand, the effective wavelengths around 468 nm could be assigned to the absorption of blue color at approximately 450 nm^35^. For silt, the significant wavelengths around 1068 and 1598 nm could be due to the absorption of soil aromatic compounds containing the C-H functional group^36^. Since the soil samples were air-dried, the important wavelengths around 1400 nm for estimating clay, sand, and silt could be related to 1^st^ overtone of the C-H functional group, relevant to soil organic matter^9^. After a close investigation of the data, a significant correlation was observed between soil texture components and organic matter of samples (data is not shown). Therefore, it could be concluded that the soil texture components (clay, sand, and silt) had indirect spectral responses and their estimation using Vis–NIR spectroscopy can be based on the co-variance with spectrally active soil components such as organic matter^1^. Finally, significant wavelengths around 641 and 651 nm for estimating clay and sand, respectively, may be assigned to absorptions caused by excitations from the ground state to the higher energy state^9^.

Detection of soil CCE from spectroscopic data was a relatively complex process. This can be due to the fact that the absorption bands of CCE shift to longer wavelengths because of the iron (Fe) impurities in dolomite, and to shorter wavelengths due to magnesium (Mg) impurities in calcite^37^. Moreover, the presence of calcium carbonate was considerable in our calcareous soil samples and it could bind with phosphor as calcium phosphate^38^. Therefore, the important wavelength of 1401 nm could be compared with 1439 nm, which is related to calcium phosphate^39^. In most studies, however, the absorption around 2300 nm was found to be closely related to the presence of carbonate^32^. Because of the NIR spectroscopic range of this research (950–1650 nm), the CCE estimations could be also related to the correlation with the other spectrally active soil parameters. Therefore, the high b-values around 1007 nm and 1401, 1512, and 1560 nm for estimating soil CCE (Fig. 3d) could be attributed to a significant correlation of CCE with soil organic matter and total soil nitrogen, respectively (data is not shown).

Finally, regarding soil EC, previous studies have shown that soil salinity estimation is controlled by association with spectrally active soil components such as water^40^. Although the samples used in this study were air-dry, soil components were still able to retain some of the soil moisture. Therefore, the high b-value around the wavelength of 1448 nm in estimating EC was likely due to the strong water absorption around 1430 nm. Other important wavelengths for estimating EC around 1040 and 1589 nm were close to wavelength bands around 1070 and 1589 nm which were identified as efficient for total nitrogen estimation^8^.

### Modeling by ANN

**Figure 4:**
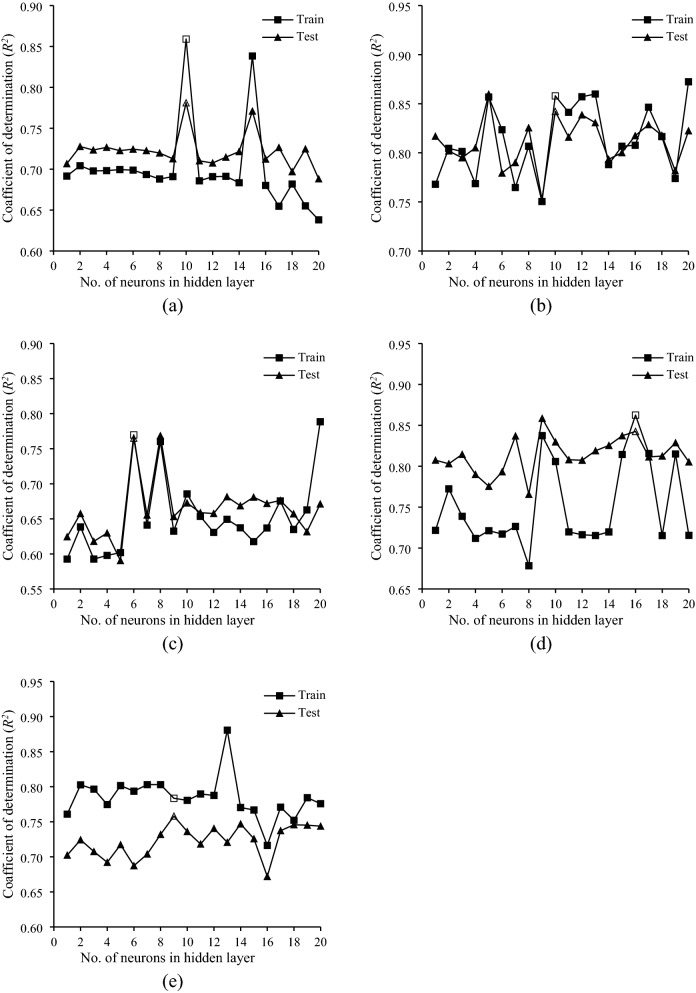
The performance of ANN models versus the number of neurons in hidden layer for estimating (**a**) clay, (**b**) sand, (**c**) silt, (**d**) calcium carbonate equivalent (CCE), and (**e**) electrical conductivity (EC).

Table 4 summarizes the results of ANN regression for predicting the selected soil physicochemical properties using spectral data of CCD and InGaAs spectrometers and the combination data of both. The prediction power (in terms of *R*^2^ values in both training and testing steps) of various models with the different number of nodes in the hidden layer (1 to 20) is shown in Fig. 4. In this figure, all ANN models were developed using the optimal spectroscopic range, resulting in the best predictability in estimating the soil physicochemical properties (Table 4). As shown, the best estimation powers and consistency (in terms of close performance in training and test steps) for predicting clay, sand, silt, CCE, and EC occurred with the optimal number of nodes of 10, 10, 6, 16, and 9 in the hidden layer, respectively.

**Table 4:** ANN calibration and estimation results of selected soil physicochemical properties using spectral data of CCD and InGaAs spectrometers, and the combination data of both.

Soil property	Spectrometer	Activation function		Training			Test set validation		
		Hidden layer	Output layer	NHL	*R*^2^_t_	RMSET	*R*^2^_p_	RMSEP	RPD
Clay (%)	CCD	Tanh	Exponential	4	0.697	2.11	0.656	2.52	1.66
	InGaAs	Logistic	Tanh	16	0.736	2.00	0.725	2.29	1.83
	Combination	Exponential	Exponential	10	0.859	1.43	0.781	1.78	2.35
Sand (%)	CCD	Logistic	Identity	6	0.792	3.32	0.778	3.47	2.13
	InGaAs	Tanh	Logistic	19	0.872	2.57	0.872	3.33	2.22
	Combination	Logistic	Tanh	10	0.858	2.85	0.842	2.81	2.63
Silt (%)	CCD	Exponential	Logistic	4	0.813	1.99	0.584	3.74	1.48
	InGaAs	Logistic	Identity	6	0.770	2.42	0.765	2.20	2.52
	Combination	Logistic	Logistic	13	0.567	3.27	0.537	3.48	1.59
CCE (%)	CCD	Tanh	Logistic	10	0.765	1.22	0.604	1.62	1.57
	InGaAs	Tanh	Exponential	16	0.863	0.96	0.843	1.45	1.76
	Combination	Logistic	Identity	7	0.896	0.81	0.554	1.70	1.50
EC (dS.m^-1^)	CCD	Exponential	Exponential	8	0.842	0.240	0.574	0.478	1.41
	InGaAs	Logistic	Tanh	9	0.784	0.328	0.758	0.303	2.22
	Combination	Logistic	Tanh	6	0.834	0.249	0.691	0.361	1.87

*CCD* charge-coupled device, *CCE* calcium carbonate equivalent, *EC* electrical conductivity, *InGaAs* indium gallium arsenide, *NHL* number of nodes in hidden layer, *R*^2^_p_ coefficient of determination in prediction, *R*^2^_t_ coefficient of determination in training, *RMSEP* root mean square error of prediction, *RMSET* root mean square error of training, *RPD* residual prediction deviation, *Tanh* hyperbolic tangent.

The best estimation among the selected properties belonged to sand (RPD = 2.63), followed by silt, clay, EC, and CCE having the RPD values of 2.52, 2.35, 2.22, and 1.76, respectively (Table 4). These RPD values could allow us excellent predictions of sand and silt, very good estimations of clay and EC, and a fair prediction of CCE, based on the criterion presented by Rossel, et al.^23^.

Moreover, utilizing the nonlinear ANN method led to a remarkable improvement in prediction powers of clay (17%), silt (5%), and EC (8%), when the RPD values of the best ANN and PLS models were compared. However, no noticeable improvement in predictability of sand (RPD of 2.63 for both ANN and PLS), and slightly less accuracy in the prediction of CCE (RPD of 1.76 and 2.01 for ANN and PLS, respectively) were obtained.

**Figure 5:**
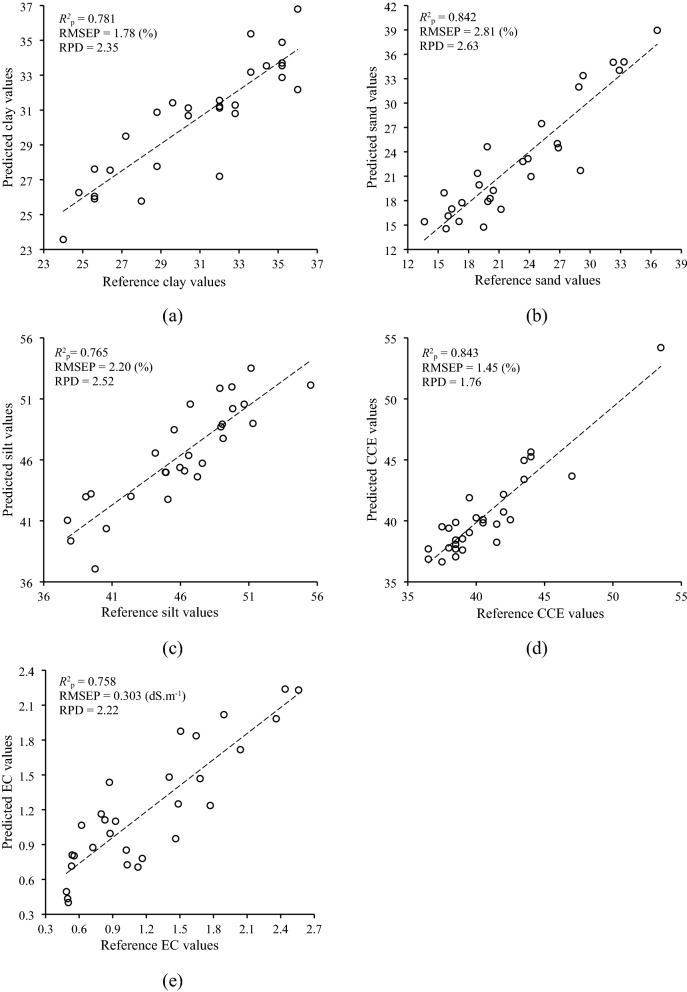
Estimated versus reference values obtained from the best ANN models of (**a**) clay and (**b**) sand using combination data of both spectrometers, and (**c**) silt, (**d**) calcium carbonate equivalent (CCE), and (**e**) electrical conductivity (EC) using spectral data of InGaAs spectrometer.

Similar to PLS, the spectral data of the InGaAs spectrometer was necessary to reach the best ANN predictive models of all selected soil properties. Figure 5 illustrates the estimated versus reference values of selected soil physicochemical properties obtained from the best ANN regression models.

Regarding the CCD instrument and in comparison with PLS results, the ANN regression could slightly enhance the predictability of clay and sand (approximately 2%). However, the predictability of the silt, CCE, and EC decreased by 17%, 6%, and 23% in using the ANN regression method (Tables 3 and 4).

### Spatial variability mapping

Table 5 summarizes the results of the best models for estimating studied soil properties using the optimum and CCD spectral data. The NIR spectral range (950–1650 nm) was essential for achieving the best predictive models of sand and silt (RPD up to 2.50, excellent performance), and clay, CCE, and EC (RPD up to 2.00, very good performance). The CCD spectroscopic instrument, however, was able to attain a very good performance for estimating the sand (RPD of 2.13), good performance for predicting EC (RPD of 1.84), and fair performance for estimating clay (RPD of 1.66), silt (RPD of 1.78), and CCE (RPD of 1.67). Despite a remarkable decrease in the predictability of clay, sand, silt, CCE, and EC by using CCD spectrometer (29.36%, 19.01%, 29.37%, 16.92%, and 17.12%, respectively, compared to the best models), the noticeable lower price of CCD spectrometer should be considered in selecting the appropriate instrument for a specific application.

**Table 5:** The results of the best models, obtained from optimum and CCD spectral data for estimating physicochemical properties of soil.

Soil properties	Spectral range (nm)	Preprocessing	Modeling methods	Model validation		
				*R*^2^_p_	RMSEP	RPD
Clay (%)	400–1650	LBC + BOE	ANN	0.871	1.78	2.35
	400–1100	1st Der	ANN	0.656	2.52	1.66
Sand (%)	400–1650	RN	ANN	0.842	2.81	2.63
	400–1100	1st Der	ANN	0.778	3.47	2.13
Silt (%)	950–1650	BOE	ANN	0.765	2.20	2.52
	400–1100	1st Der	PLS	0.732	3.12	1.78
CCE (%)	950–1650	MSC	PLS	0.876	1.27	2.01
	400–1100	2nd Der	PLS	0.681	1.53	1.67
EC (dS.m^-1^)	950–1650	MEN	ANN	0.758	0.303	2.22
	400–1100	1st Der	PLS	0.695	0.366	1.84

*ANN* artificial neural networks, *BOE* baseline offset elimination, *CCE* calcium carbonate equivalent, *EC* electrical conductivity, *LBC* linear baseline correction, *MEN* mean normalization, *MSC* multiplicative scatter correction, *PLS* partial least squares, *R*^2^_p_ coefficient of determination in prediction, *RMSEP* root mean square error of prediction, *RN* range normalization, *RPD* Residual predictive deviation, *1st Der.* first derivative, *2nd Der.* second derivative.

**Figure 6:**
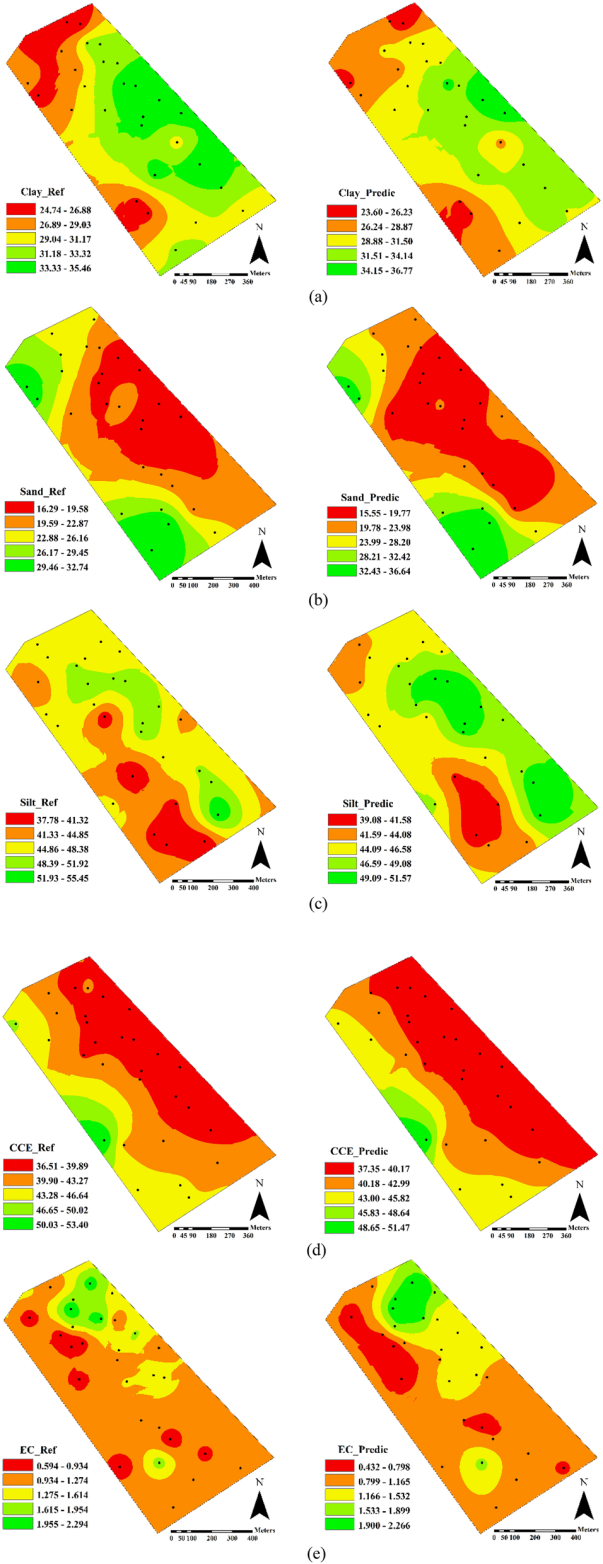
Field-scale spatial variation maps of reference and estimated values of (**a**) clay, (**b**) sand, (**c**) silt, (**d**) calcium carbonate equivalent (CCE), and (**e**) electrical conductivity (EC), obtained from best predictive models with optimum spectral range.

The spatial variability maps of soil properties obtained from the reference-measured and predicted values by the best models in the optimum spectral range are shown in Fig. 6. These maps were derived using the validation subset for each physicochemical soil property. As shown, a promising visual similarity existed in reference and predicted maps for all soil properties. About clay, the low content (northwestern corner) and high content (northeastern) areas of the studied field were obviously distinguished (Fig. 6a). Moreover, the low sand (northeastern) and high sand (south), the low silt (south) and high silt (northeastern), the low CCE (northeastern) and high CCE (southwestern), and the high EC (north) regions of the field can be clearly distinguished in predicted maps of Fig. 6b–e, respectively. Since spectral models resulted in a reliable prediction of clay, sand, silt, CCE, and EC (Table 5), there were similarities between predicted and reference maps. Therefore, the utilization of soil spectral models can be used as a layer to delineate the management zones and indirectly conduct the variable-rate fertilizer application.

**Figure 7:**
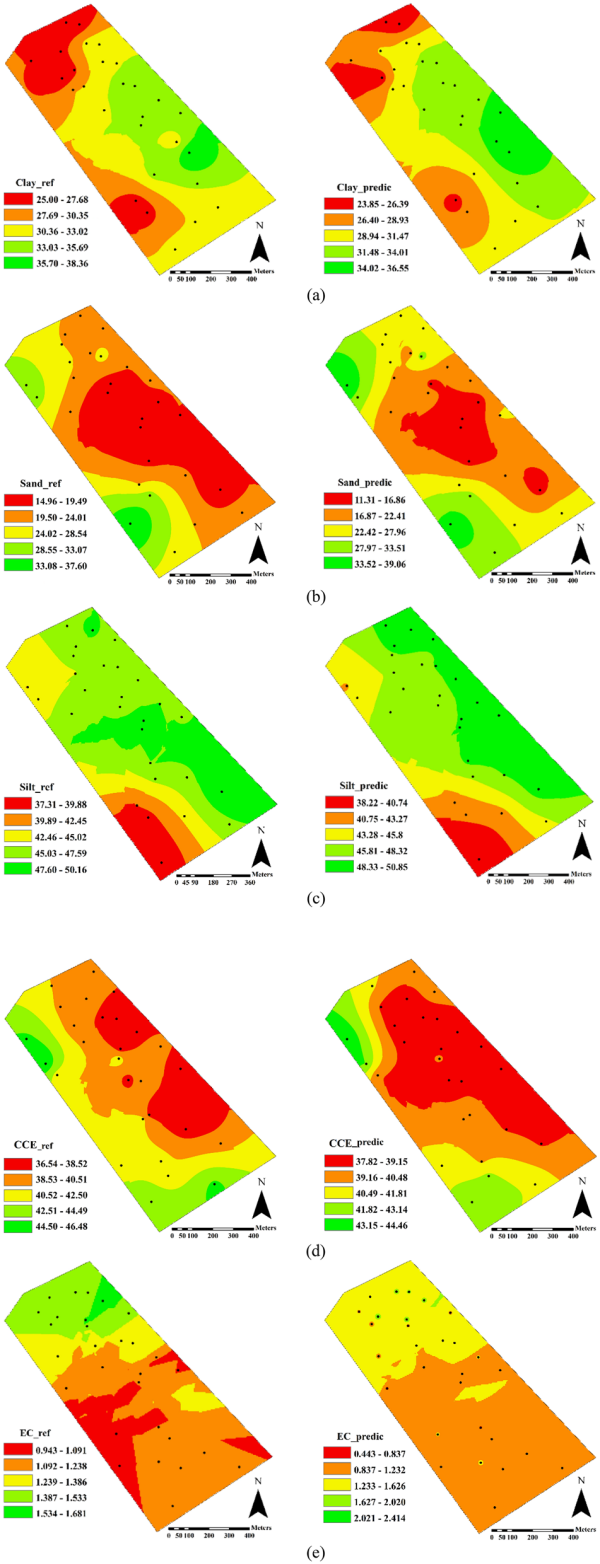
Field-scale spatial variation maps of reference and estimated values of (**a**) clay, (**b**) sand, (**c**) silt, (**d**) calcium carbonate equivalent (CCE), and (**e**) electrical conductivity (EC), obtained from best predictive models in the CCD spectral range (400–1100 nm).

The maps of laboratory reference values (test set samples) and the estimated values by the best models in the CCD spectral range (400–1100 nm) for all physicochemical soil properties are shown in Fig. 7. A visual comparison revealed a similar spatial pattern between the two maps and those obtained from the optimum spectral range (Fig. 6). The low, medium and high content zones can be distinguished for clay, sand, silt, CCE, and EC in predicted maps of Fig. 7a–e, respectively. Among the studied pairs of maps, the lowest similarity belonged to EC (Fig. 7e). As shown in Table 2, EC had the highest variation (CV of 53.43% in the test data set) among studied soil properties, and this high variance could not be successfully described by the few data points of the test set and relatively lower prediction power of corresponding Vis–SWNIR model (RPD of 1.84). Furthermore, closer spatial similarity can be observed in the variability maps of Fig. 6 (optimum spectral range) than those presented in Fig. 7 (CCD spectral range). This is due to the fact that the more accurate models were obtained by optimum spectral data (RPDs up to 2) in comparison with the CCD spectral range (RPD of 1.66–2.13) (Table 5).

The results of this study revealed that the InGaAs spectral data was mandatory to obtain the best predictive models and most accurate variability maps of all studied soil physicochemical properties. However, acceptable predictions and similar variability maps were provided via the CCD-based spectrometer. Hence, a compromise should be done between the accuracy of prediction and the cost of the spectroscopic device in selecting the adequate spectrometer. While InGaAs spectrometer provided more informative spectral data in the NIR range, its remarkably higher price and much higher dark current (thermally generated signal) compared to CCD spectrometers should be considered specifically in the field and online applications. In contrast, the CCD spectrometer with an inexpensive cost and the narrower spectral range in the NIR region could result in weaker but acceptable predictability and variability maps for all studied soil properties.

Finally, the multivariate techniques remarkably affected the predictability of different physicochemical parameters. Nonlinear ANN regression improved the prediction accuracy of soil texture parameters of clay (17%) and silt (5%) and chemical component of EC (8%) as compared to the linear PLS method. However, for the spectrally active component of CCE, better predictions were obtained by linear PLS regression for both optimum (RPD of 2.01) and CCD (RPD of 1.67) spectral data compared to ANN. It is likely due to the direct absorption of calcium phosphate and the significant correlation of CCE with the other spectrally active soil parameters, including soil organic matter and total nitrogen.

## Conclusions

This research compared the capability of two spectroscopic devices (CCD Vis/SWNIR and InGaAs NIR spectrometers) and their data combination in estimating and spatial variation mapping the selected physicochemical properties (clay, sand, silt, CCE, and EC) for a calcareous topsoil. Two different multivariate calibration techniques of PLS as linear and ANN as nonlinear approaches were used to extract the predictive models. The following conclusions can be drawn based on the results:

1. Vis–NIR spectroscopy was evaluated as an efficient method for estimating and spatial variation mapping the physicochemical properties of calcareous topsoil. Among these properties, excellent estimations were obtained for sand, and silt, and very good predictions for clay, CCE, and EC.
2. The spectral data of the InGaAs spectrometer (NIR spectral range) was necessary to reach satisfactory predictions of all selected soil properties. However, the predictive models obtained from spectral data of the low-cost CCD spectrometer could allow us the very good prediction of silt, good estimation of EC, and fair predictions of clay, silt, and CCE.
3. The nonlinear ANN regression outperformed the linear PLS method for predicting the spectrally inactive texture parameters of soil and EC. While the PLS was appropriate for estimating the spectrally active component of CCE.
4. Comparing predicted physicochemical property maps achieved from the best models in the optimum spectral range and corresponding laboratory-measured maps showed satisfactory similarities. Moreover, except for EC, the significant similarities were attained between the Vis/SWNIR-predicted maps (data of CCD spectrometer) and the equivalent reference-measured maps for all studied physicochemical properties, indicating the potential application of low-cost CCD spectrometers for detecting the variability maps of clay, sand, silt, and CCE.

## Data Availability

The datasets generated and/or analysed during the current study are not publicly available due to the commercial spectrometer used in this study (DA 7250™ NIR analyzer, Perten Instruments), and the lack of financial support from the manufacturer for the models extracted in this research, but are available from the corresponding author on reasonable request.
